# Exploring the Neural Correlates in Adopting a Realistic View: A Neural Structural and Functional Connectivity Study With Female Nurses

**DOI:** 10.3389/fnhum.2019.00197

**Published:** 2019-06-11

**Authors:** Yuichi Ogino, Hiroaki Kawamichi, Takahiro Kakeda, Shigeru Saito

**Affiliations:** ^1^Department of Anesthesiology, Gunma University Graduate School of Medicine, Maebashi, Japan; ^2^Graduate School of Human Health Sciences, Tokyo Metropolitan University, Tokyo, Japan; ^3^Department of Nursing, Faculty of Nursing, Graduate School of Nursing, Kansai University of Social Welfare, Hyogo, Japan

**Keywords:** nurse, emotion-demanding profession, realistic view, VBM, resting state fMRI

## Abstract

Empathizing leads to positive and negative consequences. To avoid empathy-induced distress, adopting a realistic view (dealing with a situation practically and efficiently independent of one’s emotional state) is important. We hypothesized that empathy-demanding professions (e.g., nursing) may require individuals to adopt a realistic view, which may demonstrate modulated neural structure and functional connectivity. We confirmed that female nurses showed a higher tendency, compared to controls, to adopt a realistic view, using the Fantasy subscale of the Interpersonal Reactivity Index (IRI; inverse scale of the realistic view). We then employed voxel-based morphometry (VBM) and resting-state functional magnetic resonance imaging (rs-fMRI) to explore the neural underpinnings related to realistic view adoption. Nurses exhibited significantly lower gray-matter volume (GMV) in the right striatum. In multiple regression analysis, only the Fantasy subscale score showed a significant positive correlation with GMV within the striatum cluster. Moreover, nurses exhibited lower functional connectivity between the right striatum and the right lateral prefrontal cortex (PFC), representing emotional regulation. These findings show that structural differences in the striatum correlated with the realistic view. Furthermore, lower functional connectivity between the striatum and lateral PFC suggests that nurses may use efficient coping strategies that may lessen the recruitment of effortful emotional regulation.

## Introduction

Given the complexity of human society, empathy, the ability to share and assess the feelings of others, is a crucial component of social interactions (Bernhardt and Singer, 2012). Although empathy can be linked to positive social behavior (empathic concern for others’ distress; Batson, 2011; de Waal, 2008), it may also lead to empathic distress, which involves aversive and self-oriented responses that result from observing another’s negative experience (Decety and Lamm, 2006). Medical professionals who are frequently exposed to emotionally-demanding situations may be especially at risk for empathic distress, resulting in empathy-induced burnout, poor health, or depression (Figley, 2002; Singer and Klimecki, 2014; Tei et al., 2014).

Nursing is a typical example of a profession that can cause high emotional burden unless the appropriate coping strategies are fostered through professional experience (Nolte et al., 2017). One of the major proposed coping strategies for alleviating empathic distress in nurses is adopting a “realistic view” (Mészáros et al., 2013), which refers to accepting one’s circumstances without being influenced by one’s emotional states, and dealing with situations in a practical, efficient, and calm manner (Brown, 1993). The realistic view in nurses was indicated to be cultivated through clinical professional experience (Acebedo-Urdiales et al., 2016; Marcinowicz et al., 2016) or their vocational motivation (Carter, 2014). These previous studies with nurses were based on interview-based investigations; there is no direct, brief-style measure of the “realistic view.”

The Fantasy subscale of the well-validated and self-reported Interpersonal Reactivity Index (IRI; Davis, 1983) is considered to measure the degree of identification with characters in fictional situations in novels and movies. Only the Fantasy subscale among the IRI subscales represents the trait of identification (Hall and Bracken, 2011; Cheetham et al., 2014). Identification refers to the experience of imaginatively perceiving oneself as transposed into the thoughts, feelings, and circumstances of a fictional character, and of merging with or being that character, entailing self-other merging processing (Cohen, 2001; Kaufman and Libby, 2012). Interestingly, the emotional arousal elicited by the Fantasy subscales’ items, measured by event-related potentials, were correlated with its score, indicating that individuals with low Fantasy subscale score elicited few emotional fluctuations (Luo et al., 2015). Individuals with lower scores in the Fantasy subscale may be able to observe the fictional character in a detached manner and recognize the situations and circumstances calmly with few emotional fluctuations, maintaining an appropriate self-other distinction with the fictional character (Decety and Jackson, 2004; Tousignant et al., 2017). This proposition would be consistent with the “realistic view” definition (Brown, 1993). Practically, adopting a “realistic view” has been applied in cognitive behavioral therapy, e.g., to treat psychotic Fantasy conditions (Beck, 1993) or psychological distress (Gilbert et al., 2018). Besides, medical professionals tend to have lower scores on the Fantasy subscale, as shown by a study that compared experienced with inexperienced veterinarians (Schoenfeld-Tacher et al., 2017) and a study of health care providers in palliative care units (Claxton-Oldfield and Banzen, 2010). Hence, we utilized the Fantasy subscale as an inverse measure to assess to what extent nurses adopt a “realistic view.”

Large studies have shown that the empathic response can be modulated by various factors such as circumstances, experiences, behavior, attributes, and professions (Singer et al., 2006; de Vignemont and Singer, 2006; Lamm et al., 2007; Xu et al., 2009; Jankowiak-Siuda et al., 2015; Decety et al., 2016). In the first fMRI study (Cheng et al., 2007) to examine empathic modulation in terms of medical professional experience, experienced physicians showed decreased activation in pain-related brain regions (insular, somatosensory, and anterior cingulate cortices) when attending to painful situations, as compared to controls, and instead showed enhanced activation in the prefrontal cortex (PFC). Subsequently, Decety et al. (2010) showed, using event-related potentials, that physicians downregulate empathic pain responses to prevent deleterious empathic distress. These two previous studies focused on task-related brain activity elicited by evoked pain. As individual experience is closely related to not only task-related brain activity but also neural structure [gray-matter volume (GMV) differences] and functional connectivity within the human brain (May, 2011; Zatorre et al., 2012; Guerra-Carrillo et al., 2014), the neural underpinnings covarying with professional experiences would be hardwired at the neural structural and resting-state brain connectivity levels, which could be investigated by voxel-based morphometry (VBM) and resting state functional magnetic resonance imaging (rs-fMRI). Thus, we hypothesized that empathy-demanding professions may require individuals to adopt a realistic view, which may have correlates in neural structure and functional connectivity. To investigate the realistic view adoption in nurses, a sample of female nurses (Nurses) and age- and sex-matched controls (Controls) completed the Fantasy subscale of the IRI. Second, participants among the IRI responders from both groups underwent VBM and resting state fMRI scanning to investigate the neural differences between the two groups.

The participants also completed the short form-36 health survey (SF-36). The SF-36 [SF-36v2^®^ Standard (Japanese), Health Survey Quality Metric Incorporated, Medical Outcomes Trust and Shunichi Fukuhara. All rights reserved] is a well-validated health-related measure used in various populations including nurses in particular (Duarte et al., 2016), providing two summary scores: the mental component summary (MCS) score and physical component summary (PCS) score (McHorney et al., 1993; Jenkinson et al., 1994; Fukuhara et al., 1998). To investigate a possible correlation between the Fantasy subscale score and GMV within a significant cluster in VBM analysis, we further conducted multiple regression analysis using the following three variables: the Fantasy Subscale, MCS, and PCS scores. We further examined whether the significant cluster would show modulated functional connectivity.

## Materials and Methods

### General Design and Participants

The general design comprised the following two components: (1) an IRI session; and (2) an MRI session. In the first, which preceded the MRI session, the female nurse group (Nurses, *n* = 43, age = 30.9 ± 8.4 years, working experience = 7.2 ± 7.3 years) and the age- and sex-matched control group (Controls, *n* = 42, age = 29.4 ± 9.6 years) completed the IRI. The MRI session followed; due to the equipment regulations in our facility, in which clinical applications are prioritized, it was not possible for all IRI session participants to undergo MRI scanning. The data from one nurse was excluded from the analysis because of a neurological disorder. We finally included the data of 20 Nurses (*n* = 20, age = 31.5 ± 8.8 years), who had 8.0 ± 8.3 years of working experience, and 20 Controls (*n* = 20, age = 31.9 ± 10.3 years). The number of subjects in our MRI sessions (*n* = 20 in each group) is considered sufficient for a neuroimaging study based on a previous review (Friston, 2012). All participants were right-handed according to the Edinburgh handedness inventory (Oldfield, 1971) and had no history of psychiatric or neurological disorders. All participants in the MRI session completed questionnaires regarding education duration, hand dominance (Oldfield, 1971), the Stanford Sleepiness Scale that examines drowsiness before the MRI scanning (a seven-grade evaluation; Hoddes et al., 1972), and the SF-36 (McHorney et al., 1993). The SF-36 is a well-validated health-related quality-of-life assessment for numerous physical and mental health conditions and includes 36 questions. The SF-36 provides two summary scores: the MCS and PCS scores, associated with mental health and physical functioning, respectively, derived from various domains that reflect mental, social, and physical aspects of daily life (McHorney et al., 1993; Jenkinson et al., 1994; Mishra et al., 2014).

Nurses, working in the operating room or the orthopedic surgery ward in Gunma University Hospital (Maebashi, Japan), voluntarily participated in this study. Controls were recruited through public postings, volunteered to participate, and they were all female; six were homemakers, nine were university students (but not majoring in nursing), and five were clerical employees (not in a medical environment). The current study was approved by the IRB of Gunma University Graduate School of Medicine (approval No. 1201). The participants provided their written informed consent and were financially compensated for their inclusion in this study.

### IRI Data Acquisition

The IRI consists of four subscales: Perspective Taking, Empathic Concern, Fantasy, and Personal Distress (Davis, 1983). Each subscale contains seven items. They are measured on a 5-point Likert scale ranging from 1 (“Does not describe me well”) to 5 (“Describes me very well”). For each subscale, a minimum score of 5 and a maximum score of 35 is possible.

For the main statistical analysis, we focused on the Fantasy subscale of the IRI, as it could be used to measure the inverse of the realistic view as described in the “Introduction” section. The Fantasy subscale includes items such as: “I daydream and fantasize, with some regularity, about things that might happen to me,” “After seeing a play or movie, I have felt as though I were one of the characters,” “I am usually objective when I watch a movie or play, and I don’t often get completely caught up in it (reversed-scored item).” Regarding the other three subscales, Empathic Concern and Personal Distress measure affective reactions; Personal Distress is self-oriented and associated to aversive emotional responses in the observer (e.g., feelings of fear or discomfort at witnessing the negative experiences of others), and Empathic Concern is other-oriented and related to feelings of compassion and sympathy for the observed individual. Perspective Taking examines the tendency to think from another’s perspective (i.e., cognitive responses; Davis, 1983).

### MRI Data Acquisition

MRI scanning was performed using a 3 T scanner (MAGNETOM Prisma 3T, Siemens, Erlangen, Germany) at Gunma University Hospital. Whole-brain, high-resolution, T1-weighted anatomical MRI using magnetization prepared rapid acquisition gradient echo (MP-RAGE) was performed for each participant [repetition time (TR) = 2,300 ms; echo time (TE) = 2.98 ms; field of view (FOV) = 256 × 256 mm^2^; flip angle = 9°; matrix size = 256 × 256 pixels; slice thickness = 1 mm; and slice number = 192]. We also performed resting state fMRI using an echo planar imaging (EPI) gradient-echo sequence (total number of volumes = 161; TR = 2,300 ms; TE = 25 ms; FOV = 220 × 220 mm^2^; flip angle = 90°; matrix size = 74 × 74 pixels; slice thickness = 3 mm; and slice number = 40). Each image was examined for artifacts.

### Data Pre-processing

For VBM, the VBM8 toolbox (revision 435) implemented in Statistical Parametric Mapping (SPM) 8 (revision 5236; The Wellcome Trust Centre for Neuroimaging^1^) was applied for the morphometric analysis (Ashburner and Friston, 2000; Ashburner, 2007). The structural images were corrected for bias-field inhomogeneity and spatially normalized with diffeomorphic anatomical registration through exponentiated Lie algebra (DARTEL) to the Montreal Neurological Institute (MNI) template, and tissues were classified as gray matter, white matter, or cerebrospinal fluid. In the modulation process, nonlinear deformation was used for normalization so that voxel intensities reflected regional GMV adjusted for individual brain sizes. Images were then smoothed to a Gaussian kernel of 8-mm full width at half maximum (FWHM).

For functional connectivity analysis, the first four volumes of each resting state fMRI run were discarded to allow for T1 equilibrium effects. After performing motion correction, we used Fourier phase-shift interpolation to correct the slice timing of each image to the middle slice. The mean of the realigned EPI images was then coregistered with the T1-weighted MP-RAGE image. Subsequently, the coregistered T1-weighted MP-RAGE image was normalized to the MNI template using linear and nonlinear three-dimensional transformations. The parameters from this normalization process were applied to each of the EPI images. Finally, the anatomically normalized EPI images were resampled to a voxel size of 2 mm × 2 mm × 2 mm and spatially smoothed using a Gaussian kernel of 8 mm FWHM. After realignment, we examined the head-movement parameters.

### Main Statistical Analysis

After pre-processing of the structural and functional images, we conducted second-level analysis (group-analysis). In the group-analysis of structural images, we conducted whole-brain analysis to investigate GMV differences between the groups, using a two-sample *t*-test, with age as “effects of no interest.” The statistical threshold of significant difference was set at false discovery rate (FDR) corrected *p* < 0.05 at the cluster level with uncorrected *p* < 0.005 at the voxel level (Lieberman and Cunningham, 2009; Slotnick, 2017; Han and Park, 2018). FDR correction controls for the expected proportion of rejected hypotheses that are false positives, different from the familywise error (FWE) that mainly controls for type I error and can dramatically increase type II errors (Nichols and Hayasaka, 2003). We set this cluster-forming threshold because previous studies have shown that the combination of FDR correction thresholding at *p* < 0.005 produced a desirable balance between the type I and II error rates on claiming significance, especially in psychological and behavioral neuroimaging analyses (Lieberman and Cunningham, 2009; Slotnick, 2017; Han and Park, 2018). To examine for a possible relationship between the Fantasy subscale score and GMV within clusters, we also conducted multiple regression analysis using the following three variables: the Fantasy Subscale, MCS scores, and PCS scores, with the average beta value within significant clusters as the dependent variable.

We then conducted a functional connectivity analysis using the CONN toolbox (version 15.h) on SPM8 (revision 5236). In the functional connectivity analysis, we set the right striatum cluster as the seed region, based on the regions showing significant difference in the VBM analysis. By using the CONN toolbox, we conducted a seed-driven functional connectivity analysis (Seed-to-Voxel analysis) in which Pearson’s correlation coefficient is calculated between the seed time course and the time course of all other voxels (Whitfield-Gabrieli and Nieto-Castanon, 2012). The correlation coefficients are then converted to normally distributed scores using Fisher’s transformation to allow for second-level general linear model analysis, using the same significance threshold (FDR corrected *p* < 0.05 at the cluster level with uncorrected *p* < 0.005 at the voxel level). Differences between the groups were assessed by *t*-test. Data are presented as means ± standard deviation (SD) unless otherwise indicated.

## Results

### IRI Scores and Demographics of Participants

Table 1 shows the scores on each IRI subscale for participants in the IRI session (Nurses: *n* = 42, age: 30.9 ± 8.4 years; Controls: *n* = 42, age: 29.4 ± 9.6 years). Nurses had a significantly lower score than that of the Controls (*p* = 0.028) only in the Fantasy subscale in the IRI session (Table 1).

**Table 1:** Interpersonal Reactivity Index (IRI) scores of the participants.

IRI Subscale	Perspective taking	Empathic concern	Fantasy	Personal distress
Nurses	16.6 ± 3.3	16.8 ± 3.1	15.6 ± 3.9	14.8 ± 2.9
Controls	17.0 ± 2.9	17.3 ± 1.8	18.0 ± 4.2	14.2 ± 3.8
*t*-test	*p* = 0.656	*p* = 0.524	*p* = 0.028*	*p* = 0.506

All participants were female. In the IRI session, we included Nurses (n = 42; Age: 30.9 ± 8.4 years) and Controls (*n* = 42; Age: 29.4 ± 9.6 years). The IRI contains four subscales: Perspective taking, Empathic concern, Fantasy, and Personal distress. There was a significant difference between Nurses and Controls only in the Fantasy subscale score (*p* = 0.028*). Data are expressed as means ± standard deviation (SD). *p < 0.05 using two-sample *t*-test. n = number of subjects.

In Table 2, we present the comparative demographics of the participants in the MRI session (Nurses: *n* = 20; Controls: *n* = 20). There was no significant difference between the groups in age, handedness (all right-handed), Stanford Sleepiness Scale before the MRI scanning, education duration, or MCS and PCS scores. There were no significant differences in the Fantasy subscale scores between the Nurses and Controls that participated in the MRI sessions (*p* = 0.131; Table 2). We confirmed that there was no difference in age (*p* = 0.633) and Fantasy subscale scores in both Nurses (*p* = 0.501) and Controls (*p* = 0.401) between sessions (IRI and MRI sessions). We also confirmed that there was no difference in working experience (*p* = 0.687) between the Nurses in the IRI session (86.2 ± 87.6 months) and those in the MRI session (96.4 ± 100.1 months). As we found a clear significant difference in the Fantasy subscale score between the groups in the IRI session (Table 1), we proceeded to examine the correlation between the Fantasy subscale score and GMV within a significant cluster in multiple regression analysis as described below.

**Table 2:** Comparative demographics of the participants in the MRI session.

MRI session	Nurses	Controls	*p* value
Age	31.5 ± 8.8	31.9 ± 10.3	0.972
Handedness	37.6 ± 4.0	37.8 ± 2.2	0.846
Educational duration (years)	15.56 ± 1.5	16.67 ± 2.2	0.170
Stanford Sleepiness Scale Score	2.35 ± 0.7	2.30 ± 0.6	0.813
Fantasy subscale score	15.05 ± 3.9	16.80 ± 4.2	0.131
MCS score	50.19 ± 7.3	49.91 ± 6.6	0.901
PCS score	55.66 ± 6.1	57.41 ± 6.5	0.383

*Twenty nurses and 20 controls among those who participated in the IRI session underwent MRI scanning. Differences between the groups were assessed by *t*-test. We confirmed that there was no difference between sessions (IRI and MRI sessions) in age, Fantasy subscale score, and education duration in both groups. MRI, magnetic resonance imaging; IRI, Interpersonal Reactivity Index; n, number of subjects; MCS, Mental component summary; PCS, Physical component summary*.

### MRI (VBM and Functional Connectivity Analysis)

**Figure 1:**
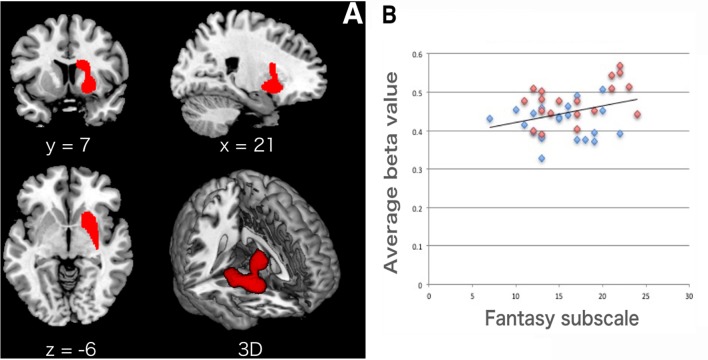
**(A)** Lower gray matter volume (GMV) in the right striatum in Nurses. The location of a significant cluster in the right striatum (from the putamen to the caudate nucleus) is presented in the x, y, and z axes and in 3D. The statistical threshold for significant differences was set at false discovery rate (FDR) corrected *p* < 0.05 at the cluster level with uncorrected *p* < 0.005 at the voxel level. **(B)** Significant correlation between the Fantasy subscale score and the striatum. We found a significant correlation between the Fantasy subscale and the average beta value (GMV) within the significant striatum cluster [*R* = 0.384 (*p* = 0.046)]. The cluster average beta value was calculated using MarsBaR (http://marsbar.sourceforge.net). Blue dots, Nurses; red dots, Controls.

In the whole brain VBM analysis in the MRI session (Nurses: *n* = 20; Controls: *n* = 20), Nurses showed significantly lower GMV in the right striatum [ventral and dorsal part of the striatum including the head of the caudate as shown in Figure 1A; top peak = (21, 2, −6); FDR-corrected *p* = 0.011; number of voxels = 2,164] as shown in Table 3. We did not find any other significant clusters between the groups. Forward step model multiple regression analysis using the Fantasy subscale, MCS, and PCS scores showed that only the Fantasy subscale score had a significant positive association with the average beta value in the right striatum cluster (*p* = 0.046; Table 4). We also confirmed that there was no multicollinearity among the variables (Fantasy subscale, MCS, and PCS scores). Figure 1B illustrates a significant correlation between the Fantasy subscale score and the average beta value in the significant striatum cluster [*R* = 0.384 (*p* = 0.046)].

**Table 3:** Significant difference in gray matter volume (GMV) between the groups (Control > Nurse).

	Cluster *p* (FDR)	Cluster size	*x*	*y*	*z*	*t*-value
			21	2	−6	3.64
Right striatum	0.011	2,164	30	−10	−8	3.57
			12	8	24	3.52

*Coordinates refer to local cluster maxima. FDR corrected p < 0.05 at the cluster level with uncorrected *p* < 0.005 at the voxel level were employed. FDR, false discovery rate*.

**Table 4:** Multiple regression analysis using the average beta value in the striatum cluster as the dependent variable in the MRI session.

			95% CI	
Variables	*β* value	*p* value	lower	upper
Fantasy subscale	0.0042	0.0459*	0.0001	0.0084
MCS score	−0.0013	0.2932	−0.0038	0.0012
PCS score	0.0011	0.4236	−0.0016	0.0038

In multiple regression analysis, only the Fantasy subscale score showed a significant positive association with the average beta value in the striatum cluster (*β* = 0.0042, *p* = 0.0459). *p value less than 0.05 was considered statistically significant. MRI, magnetic resonance imaging; MCS, Mental component summary; PCS, Physical component summary; CI, confidence interval.

**Figure 2:**
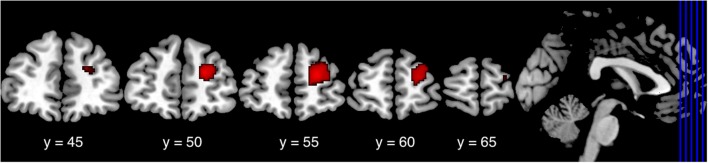
Lower connectivity in the right lateral PFC cluster in Nurses. In functional connectivity analysis seeding the right striatum cluster, Nurses showed significantly lower functional connectivity between the right lateral PFC and the right striatum (cluster FDR *p* = 0.048) than did the Controls. Lateral PFC, lateral prefrontal cortex; FDR, false discovery rate.

To investigate differences in functional connectivity with the striatum, we further analyzed the resting state fMRI data of Nurses and Controls with the right striatal cluster as a seed region. As a result, we found significantly lower functional connectivity between the right lateral PFC and the right striatum in Nurses (cluster FDR *p* = 0.048; Table 5, Figure 2). We did not find any other significant differences between the groups in terms of functional connectivity.

**Table 5:** Significant brain regions seeding the right striatum between the groups (Control > Nurse) in the resting state fMRI analysis.

	Peak *p* (FDR)	Cluster size	*x*	*y*	*z*	*t*-value
Right lateral PFC	0.048	383	22	52	18	4.42

*Coordinates refer to local cluster maxima. The statistical threshold for significant differences was set at FDR corrected *p* < 0.05 at the cluster level with uncorrected *p* < 0.005 at the voxel level. FDR, false discovery rate; resting state fMRI, resting-state functional magnetic resonance imaging; PFC, prefrontal cortex*.

## Discussion

Although nursing is a typical empathy-demanding profession (Carter, 2014; Acebedo-Urdiales et al., 2016; Nolte et al., 2017), nurses ordinarily care for patients properly and efficiently. In this sense, it is of great importance to elucidate the neural mechanisms implicated in realistic view adoption, which would control the empathic distress that nurses would face directly in their professional circumstances and experiences. Our main findings comprise the following two results: (1) the Nurses had significantly lower Fantasy subscale scores, indicating a higher tendency to adopt a realistic view. The Nurses showed significantly lower GMV in the right striatum. In multiple regression analysis, we identified a significant positive correlation between the Fantasy subscale score and the averaged beta value in the right striatum cluster (i.e., negative correlation between the realistic view and the GMV within the striatal cluster); and (2) the Nurses showed significantly lower functional connectivity between the right striatal cluster and the right lateral PFC.

### Lower GMV in the Striatum in Nurses

It is well known that the striatum is involved in multiple and heterogeneous functions, including in motor action and motivational, cognitive, and emotional processing (Di Martino et al., 2008; Graybiel and Grafton, 2015; Jarbo and Verstynen, 2015). The Fantasy subscale score is also known to be associated with striatal activity and morphology as follows: in terms of pathological findings accompanying high Fantasy subscale scores, the Fantasy subscale was reported as a unique positive predictor of awareness of mental illness (Atoui et al., 2018). Among the IRI subscales, only the Fantasy subscale was associated with an increased risk of delusions (Montag et al., 2012) and obsessive-compulsive disorder (Fontenelle et al., 2009). Patients with delusions who spend excessive amounts of time engaged in mental fantasy worlds showed considerable activity in the striatum in an fMRI study (Bigelsen et al., 2016) as well as erotic fantasizing (Brand et al., 2016). Psychopathic personality (Glenn et al., 2010; Vieira et al., 2015) as well as impulsivity (Tschernegg et al., 2015) were associated with increased GMV in the striatum. It is sometimes assumed that cortical morphology is positively associated with trait behavior (Gardini et al., 2009; DeYoung et al., 2010). As the Fantasy subscale score is reportedly an inverse measure of the realistic view, it is thus conceivable to consider that the realistic view could be associated with striatal activity and morphology, although the association remains unexamined.

Striatal activity is modulated by the various contexts in our complex environment including our profession and relationships (Engelmann and Hein, 2013). Evidence from neuroplasticity studies has suggested that environmental demands could result in significant changes in the representative neural structures that underlie skills such as those employed by jugglers (Draganski et al., 2004), musicians (Münte et al., 2002), and bilinguals (Li et al., 2014). Although most of these brain imaging studies have reported that enlargement of the involved brain regions results from their intense and repeated activation, successful chess players, who required the realistic view (to maintain practical and efficient judgment without being influenced by emotional fluctuation; Hernández Hernández and Rodríguez-Mateo, 2006), consistently exhibited lower GMV in the striatum (Duan et al., 2012; Hänggi et al., 2014). Similarly, in terms of striatal modulation by social human relationships (Engelmann and Hein, 2013), our recent study also showed that being in a romantic relationship was associated with significantly lower GMV in the right dorsal striatum and increased subjective happiness (Kawamichi et al., 2016). Therefore, we suggest that the current findings that the Nurses showed lower GMV in the right striatum negatively correlated with the realistic view, might be a representation of morphological modulation due to their professional circumstances and experiences. We consider the realistic view as one of the important representations of striatal function, along with the motor, motivational, cognitive, and emotional functions.

### Lower Connectivity Between the Striatum and the Lateral PFC in Nurses

Emotional regulation is important for empathic modulation (Decety and Lamm, 2006; Decety et al., 2010; Engen and Singer, 2013). Although both the medial and lateral PFCs are involved in emotional regulation (Etkin et al., 2015), the lateral PFC has been traditionally considered the key region of negative emotion regulation through cognitive reappraisal (Ochsner and Gross, 2005; Ochsner et al., 2012; Etkin et al., 2015). As the enhancement (upregulation) of negative emotions is left-lateralized and inhibition (downregulation) involves both sides of the lateral PFC (Ochsner et al., 2004; Kim and Hamann, 2007; Wager et al., 2008), the lateralization of the functional connectivity between the right lateral PFC and the right striatum is likely related to emotional regulation, especially inhibition of negative emotion.

The classical concept of emotional regulation includes the following two different processes: “cognitive reappraisal” and “expressive suppression” (Gross, 1998). Cognitive reappraisal is defined as the attempt to reinterpret an emotion-eliciting situation in a manner that alters its meaning and decreases its emotional impact, and expressive suppression is defined as the attempt to hide or inhibit outward emotional expression of inner feelings (Gross, 1998). The distinction between these two emotion regulations lies on cognitive cost and memory. In emotional events, cognitive reappraisal causes less psychological burden than does expressive suppression and has no impact on the memory of events, while expressive suppression impairs memory (Richards and Gross, 2000; Gross, 2002; Cutuli, 2014). Cutuli (2014) proposed that cognitive reappraisal is often more effective and healthier than expressive suppression. As Tei et al.’s (2014) study showed, expressive suppression (discrepancy between inner feelings and outer expression) is correlated with burnout severity in medical professionals. Therefore, the cognitive cost of the realistic view expressed as “keeping one’s cool” in Richards and Gross’s (2000) study may vary, depending on how these two strategies are selected and used according to the situations and circumstances (Gross, 2002; Cutuli, 2014).

Our suggestion is that the Nurses’ realistic view serves as an efficient reappraisal strategy in daily practice. For example, Hallam et al. (2015) showed that realistic and efficient reappraisal toward emotion-eliciting events (i.e., realistic view) evoked less PFC activation compared to effortful reappraisal. Lewis et al. (2009) demonstrated that learning and experience change resting functional connectivity. Thus, the realistic view in the Nurses may entail less recruitment of effortful emotional regulation and may thereby lead to lower functional connectivity between the striatum and the lateral PFC at the resting state.

Finally, there were apparent discrepancies between our results and those of Cheng et al. (2007). In Cheng et al.’s (2007) study, experienced physicians showed medial and dorsolateral PFC activation, implying emotional regulation (inhibition) derived from painful stimulation. In contrast, our resting-state functional connectivity analysis revealed that the Nurses showed lower functional connectivity between the right lateral PFC and the right striatum. The difference could be attributable to methodological differences between Cheng et al.’s (2007) study and ours. Cheng et al.’s (2007) used event-related fMRI with painful stimuli, which evoked emotion explicitly and required immediate inhibitory emotional regulation. In contrast, the present study investigated functional connectivity during the resting state, which reflects brain activity related to the default mode. As mentioned above, the cognitive cost of emotional regulation depends on how the two strategies (“cognitive reappraisal” and “expressive suppression”) are selected. Thus, humans select one of the two strategies according to situations and circumstances (Richards and Gross, 2000; Gross, 2002; Cutuli, 2014). While cognitive reappraisal is an attempt to reinterpret the situation before the complete activation of the emotional response, expressive suppression is a response-focused strategy that intervenes toward the complete emotional response evoked by the emotion-eliciting situation (Cutuli, 2014). As the medical professionals in Cheng et al.’s (2007) study were exposed to the experimental, explicitly emotion-eliciting painful condition, they may have adopted expressive suppression for inhibitory emotional regulation, focusing on the evoked emotional response. A subsequent study (Decety et al., 2010) furthermore showed that inhibitory emotional regulation (evoked by explicit emotion-eliciting painful stimuli) occurred at an early stage in the emotion-generative process in medical professionals, and they suggested that such an early inhibitory effect might be beneficial for medical professionals confronting severe emotion-demanding events. In sum, we therefore consider that Cheng et al. (2007) and our results are not contradictory, but the reflection of the differences of the situations and circumstances that medical professionals confront, stemming from differences in methodological approaches between Cheng et al.’s (2007) study and ours. For balancing cognitive cost and immediate effect, medical professionals (Nurses in the present study) may select and use these two strategies as dictated by the situations and circumstances at hand. Future studies should further investigate such adaptation by evaluating functional connectivity with and activities in the PFC in the same sample.

### Limitations

The present study had a cross-sectional design; thus, the question of whether the anatomical changes are the cause or consequence of professional experience remains unanswered, as the structural differences could be attributable to an innate predisposition. This question could only be answered by a longitudinal study that would follow new nursing staff through their career to clarify the causal relationship between the realistic view and professional experience.

We focused on only female participants in the present study. This is because women were found to have higher expressivity in emotional responses than did men (Bianchin and Angrilli, 2012; Deng et al., 2016); thus, we determined women as being more appropriate participants than men for detecting brain activities related to emotional responses such as cognitive reappraisal and expressive suppression. However, as previous findings have shown that women scored higher on the Fantasy subscale than did men (Gilet et al., 2013; Lucas-Molina et al., 2017) and it is also suggested that there are sex differences ranging from the cultural and social levels to the phylogenetic and ontogenetic levels (Christov-Moore et al., 2014; Jankowiak-Siuda et al., 2015), a future study should also include male participants.

### In Summary

We demonstrated that Nurses tended to adopt a realistic view and that this was reflected in their lower GMV in the right striatum compared to Controls. The lower functional connectivity observed between the right striatum and the right lateral PFC in Nurses suggests that cognitive reappraisal accompanying the realistic view in Nurses may lessen the recruitment of effortful emotional regulation as an effective coping strategy. The observation that Nurses tended to adopt a realistic view signifies that empathic modulation can be beneficial for persons working in healthcare professions and may enhance the understanding of neural coping in excessively empathy-demanding circumstances.

## Ethics Statement

The current study was approved by the IRB of Gunma University Graduate School of Medicine (approval No. 1201). Nurses, working in the operating room or orthopedic surgery ward in Gunma University Hospital (Maebashi, Japan), voluntarily participated in this study. Controls were recruited through public postings and volunteered for participation. The participants provided their written informed consent and were financially compensated for their inclusion in this study.

## Author Contributions

YO and HK designed the experiments, conducted the experiments, analyzed the data, and wrote the manuscript. TK helped conduct the experiments and discuss the data. SS supervised the overall project and edited the manuscript.

## Conflict of Interest Statement

The authors declare that the research was conducted in the absence of any commercial or financial relationships that could be construed as a potential conflict of interest.

## Footnotes

^1^http://www.fil.ion.ucl.ac.uk/spm
